# Development of a High-throughput Morphological Assay for Evaluating Mesenchymal Stromal Cell-derived Extracellular Vesicle Modulation of Brain Pericyte Secretory Phenotype

**DOI:** 10.1007/s12015-025-10940-6

**Published:** 2025-08-12

**Authors:** Courtney E. Campagna, Andrew M. Larey, Kanupriya R. Daga, Morgan Roos, Sneha Ghosh, Neil Grimsey, Jin Han, Ross A. Marklein

**Affiliations:** 1 School of Chemical, Materials, and Biomedical Engineering, University of Georgia, Athens, GA USA; 2 Regenerative Bioscience Center, University of Georgia, Athens, GA USA; 3 College of Pharmacy, University of Georgia, Athens, GA USA; 4 Center for Biologics Evaluation and Research, Food and Drug Administration, 10903 New Hampshire Ave, Silver Spring, MD USA

**Keywords:** *MSC-EVs*, Pericytes, Neuroinflammation, Morphology, Secretome

## Abstract

Mesenchymal stromal cell-derived extracellular vesicles (MSC-EVs) are a promising therapeutic tool for treating many neurodegenerative diseases. Neuroinflammation plays a major role in many of these conditions through an orchestration of interdependent processes that lead to the breakdown of the blood-brain barrier (BBB), infiltration of immune cells and neuronal death. MSC-EVs have shown preliminary evidence of modulating neuroinflammation, but their mechanisms of action are still unknown. Therefore, we explored the potential of MSC-EVs in modulating brain pericytes, a cell type that plays a critical role in BBB maintenance but has not been investigated as a therapeutic target for MSC-EVs. Brain pericytes are multifaceted cells that can modulate neuroinflammation through their involvement in BBB homeostasis, as well as the innate and adaptive immune response. Pericyte morphology has been shown to change in response to inflammatory stimuli in vivo, hence, we used this behavior to develop a quantitative morphological profiling approach to assess the immunomodulatory function of MSC-EVs in a high-throughput, low-cost manner. Using this assay, we were able to demonstrate that MSC-EVs manufactured under various conditions (2D, 3D, and in response to cytokine priming) could induce distinct pericyte morphological responses indicative of changes in secretion of chemokines and cytokines relevant to neuroinflammation.

**Supplementary Information:**

The online version contains supplementary material available at 10.1007/s12015-025-10940-6.

## Introduction

Neurodegenerative diseases lead to the loss of cognitive and functional abilities due to neuronal death. Despite varying underlying causes, neuroinflammation is a common factor across many of these conditions, posing a major challenge for therapy development [1, 2]. Neuroinflammation is an immune response within the central nervous system (CNS), involving interactions between leukocytes, microglia, astrocytes, pericytes, glial cells, and their associated signaling molecules [3]. In homeostatic conditions, neuroinflammation can serve a beneficial role to protect and promote normal brain function by regulating the recognition, trafficking, and elimination of pathogens and cell debris [4]​. However, when neuroinflammation becomes chronic or dysregulated, it can result in tissue damage and further progressing disease pathology [3]. Some detrimental aspects of neuroinflammation include the release of pro-inflammatory cytokines and chemokines, activation of immune cells, the breakdown of the vasculature, and neuronal death​ [4]​. These adverse effects are often associated with the breakdown of the blood-brain barrier (BBB), the interface between the blood and the brain parenchyma that maintains the CNS stability. The BBB, composed of pericytes, astrocytes, and brain endothelial cells (BMECs), plays a key role in maintaining CNS homeostasis by regulating the passage of molecules across the blood-brain interface​ [5]​. Therefore, preserving BBB integrity, and regulating neuroinflammation necessitates an immunomodulatory, multi-cellular approach to prevent further disease progression.

Mesenchymal stromal cells (MSCs) have been explored as a therapeutic for inflammatory diseases due to their immunomodulatory behavior. In terms of neuroinflammation, MSCs have shown promise in treating neurodegenerative diseases such as traumatic brain injury (TBI) and stroke in animal models [6, 7]. However, due to the tightly regulated nature of the BBB, MSCs cannot readily enter the brain parenchyma when administered intravenously with most of injected MSCs becoming trapped in the lung [8, 9]. Additionally, local intracerebral injection of MSCs comes with inherent risks of damaging adjacent healthy tissues, even with efficient delivery of the cells [8]. Moreover, heterogeneity of MSCs has also posed challenges in comprehending both their mechanism of action and any associated safety concerns.

To address some of the challenges associated with MSCs, MSC-derived extracellular vesicles (MSC-EVs) have garnered significant interest in treating neuroinflammation. MSC-EVs are lipid-membrane-bound structures secreted by MSCs that contain bioactive molecules such as proteins, metabolites, lipids, and nucleic acids [8]​. MSC-EVs are non-replicative and small sized (~ 50–1000 s nm in diameter), posing minimal inherent risks of tumorigenicity or thrombosis [10, 11], and can readily cross the BBB [12]. Importantly, MSC-EV cargo, which includes tetraspanins, receptors, integrins, lipids, and miRNA, exhibits immunomodulatory functions similar to those of MSCs [13, 14]. Moreover, MSC-EV immunomodulatory function can also be enhanced by exposing the source MSCs to inflammatory-relevant signals (e.g. cytokines, hypoxia or low pH) for a short period of time (1–3 days), termed ‘priming’ [13]. For example, MSCs primed with reduced serum and 1% oxygen for 48 hours enhanced secretion of proteins with mitogenic and neurotrophic functions​ [15]​. Another study showed that cytokine-primed MSCs cultured in hypoxic condition further impacted lipid composition of MSC-EVs and enhanced their functionality to modulate microglia morphology [2]. While these studies provided promising preliminary evidence on the effects of manufacturing (e.g. priming) on MSC-EVs in the context of neuroinflammation, there is currently no standardized method for evaluating their immunomodulatory function. The current recommendations of the International Society of Extracellular Vesicles (ISEV) focus primarily on EV identification with no specific recommendations regarding functionality or immunomodulatory activity [16]​. Although some studies have employed multi-omics approaches and wound healing assays both in vitro and in vivo to assess EV function [17–20], these methods are often costly, low-throughput, difficult to standardize, and not specifically tailored to neuroinflammation.

Pericytes are multipotent cells [21, 22] located throughout the basement membrane of the vasculature. They are heterogeneous in origin, tissue distribution, morphology, and play a critical role in the progression of neuroinflammation due to their regulation of the BBB, as well as proximity [23]. Pericytes assist in the regulation of vasculature including angiogenesis and capillary blood flow, as well as the regeneration of function and structure in damaged parts of the CNS​ [23]. Additionally, they regulate various aspects of the immune response, such as leukocyte extravasation, inflammation-induced BBB disruption, propagation of peripheral and central inflammation, and polarization of the inflammatory cells in the BBB and the brain parenchyma​ [24]​. These diverse functions make pericytes a strong candidate target for studying neuroinflammation and for screening potential therapies. However, while they have been more actively studied as a target for drug therapies [25], the effects of MS[[C-EVs on this versatile cell type remain largely unknown. In vivo, pericytes undergo morphological changes in response to inflammation, becoming activated and often migrating away from the sites of chronic inflammation. For instance, following penetrating cortical injury in humans, several morphological types of pericytes were observed near injury site, migrating away from blood vessels [26]. Interestingly, MSCs also respond to inflammatory stimuli (in the form of cytokine priming) through morphological changes similar to pericytes. We have previously shown this morphological shift can predict a given MSC batch’s immunomodulatory function, and help screen priming conditions to optimize MSC manufacturing [27, 28]. For example, a morphological screening of MSCs primed with various combinations of Interferon-gamma (IFN-γ) and Tumor Necrosis Factor-alpha (TNF-α) identified optimal IFN-γ/TNF-α priming conditions that enhanced MSC function in terms of T cell suppression​ [28]​.

Previous works suggested pericytes and MSCs exhibit phenotypic similarities, such as cellular morphology and cell surface molecules [29, 30]. Hence, we hypothesize that a similar morphological profiling approach with pericytes could serve as an indicator of their response to inflammation and immunomodulatory effects of MSC-EV [27]. Here, we developed a high content imaging-based morphological assay on pericytes to assess the effect of MSC-EVs generated under varying manufacturing conditions (e.g., flask vs. bioreactor, priming, microcarrier density). TNF-α was used as an inflammatory stimuli to activate pericytes, as it plays a key role in neuroinflammation and many neurodegenerative diseases, including Alzheimer’s Disease, Ischemic stroke, Traumatic Brain Injury [31–33]. We present a comprehensive single-cell morphological profiling of pericyte responses to TNF-α and MSC-EVs from various manufacturing conditions. We further explore the relationship between these pericyte morphological responses and secretome profiles of cytokines and chemokines.

## Materials and Methods

### Pericyte Expansion and Cryopreservation

Human Brain Pericytes (ScienCell) were expanded in Pericyte Complete Medium (ScienCell) on T175 flasks at 3500 cells/cm^2^ coated with 1% (w/w) Poly-d-lysine (PDL) (Sigma) for four passages with TrypLE (Gibco) used to harvest cells at the end of each passage upon reaching 80–90% confluency. At passage 4, pericytes were cryopreserved in pericyte media containing 10% DMSO.

### MSC-EV Manufacturing

Human bone marrow derived MSC line RB71 was expanded according to the RoosterBio protocol previously described [28] and then cryopreserved at passage 2. One frozen vial containing 10^6^ MSCs was seeded into T225 flasks at a seeding density of 4,444 cells/cm^2^ in RoosterNourish-MSC-XF and cultured to 80% confluency. For flask MSC-EV manufacturing, the MSCs were passaged into T175 flasks at a seeding density of 714 cells/cm^2^. For the bioreactor group, MSCs were passaged into 0.5 L spinning wheel bioreactors (PBS Biotech) with 0.4 g Corning Synthemax II polystyrene microcarriers. These MSCs were expanded in their respective vessels following protocols from PBS Biotech and RoosterBio. The MSCs were then primed in serum-free RoosterCollect-EV medium with control and priming conditions. The MSC-EVs were collected from the supernatant using an adapted 2-step ultracentrifuge protocol [34]. The supernatant was first filtered through a 0.2 μm filter and then centrifuged at 133,900 xg (Soorvall WX ultracentrifuge, ThermoFisher; Fiberlite F37L-8 × 100 Fixed-Angle Rotor, ThermoFisher, k factor = 224; PC Bottle Assembly 70mL, ThermoFisher) for 1 h at 4 °C. The MSC-EV pellet was then resuspended in cold PBS-/- and then centrifuged again in micro-ultracentrifuge tubes (PC Thickwall 4mL, ThermoFisher) at 140,000xg (Sorvall MX 150 + Micro-Ultracentrifuge, ThermoFisher; S110-AT Fixed-Angle Rotor, ThermoFisher, k factor = 76) for 1 h at 4 °C. The EV pellets were then resuspended at a 37.5X concentration in cold PBS-/-. The prepared EVs were then stored at −80 °C until used.

### Pericyte Morphological Assay

A T175 flask was coated with 1% (w/w) poly-d-lysine (PDL) for 24 h at 4 °C. After, the flask was washed with PBS three times to get rid of excess PDL and 35 mL of pericyte medium (Sciencell) was added. 10^6^ previously frozen human brain pericytes (Sciencell) were thawed and seeded on the coated T175 flask. A flat-bottomed 96-well plate (Corning, Cat # 3599) was coated with 1% (w/w) poly-d-lysine (PDL) for 24 h at 4 °C. Once the pericytes reached 80–90% confluency (3 days) they were harvested using TrypLE (Gibco) and were seeded at 2,500 cells/cm^2^ for 24 h in 100 µL of pericyte complete medium in the prepared 96-well plate. After 24 h, 50% of the media (50 µL) was aspirated from each well. Then 50 µL of pericyte medium only (‘unstimulated’) or 50 µL of pericyte medium containing 50 ng/mL TNF-α (Gibco, PHC3015) (‘stimulated’) were added to appropriate wells and pericytes incubated for an additional 24 h. For MSC-EV treated pericyte groups, MSC-EVs were added concurrently with 100 ng/mL of TNF-α (50 ng/mL final concentration due to half media change) in pericyte media to the appropriate wells. We fixed samples using 4% paraformaldehyde (Electron Microscopy Sciences) and stained with Hoechst [10 µg/mL] (Invitrogen) and Fluorescein maleimide [20µM] (ThermoFisher) in PBS-/- (Gibco) for nuclear and cytoplasm morphology, respectively as done previously [27]. Pericytes were imaged using Cytation 5 High Content Imaging system (Agilent) and a Ti-Eclipse (Nikon) with the imaging system indicated in each figure legend. We imaged approximately 50% of every well using a 6 × 6 montage at 10X magnification. These images were processed on a single-cell basis for over 96 different nuclear and cytoplasmic morphological features using CellProfiler pipeline [35] (Supplementary File 1), however, we focused primarily on the 21 features [27]. A table summarizing and defining all morphological features quantified in this study can be found in Supplementary Table 1.

### Pericyte Secretome

Pericytes were cultured on a PDL coated 96-well flat-bottomed plate at a density of 22,580 cells/cm^2^ for 24 h in pericyte media. After 24 h, 50% of the media (50 µL) was aspirated from each well. Then 50 µL of pericyte medium only (‘unstimulated’) or 50 µL of pericyte medium containing 50 ng/mL TNF-α (Gibco, PHC3015) (‘stimulated’) were added to appropriate wells and pericytes incubated for an additional 24 h. For MSC-EV treated pericyte groups, MSC-EVs were added concurrently with 100 ng/mL of TNF-α (50 ng/mL final concentration due to half media change) in pericyte media to the appropriate wells. Pericyte conditioned medium was collected from *n* = 3 wells per experimental group and stored at −80 °C. Frozen CM samples were shipped on dry ice to RayBiotech (Norcross, GA) for secretome analysis using the 200plex quantibody Array (Q4000). Differentially secreted proteins of interest were analyzed using STRING database (https://string-db.org/) to identify significantly enriched pathways.

### Data Analysis and Statistics

Singe cell morphological data is presented on a median-per-well basis and summarizes data from at least 300 cells per well. All data and statistical analyses were performed using GraphPad Prism v10 with specific statistical tests performed are detailed in each figure legend.

## Results

### TNF-α-stimulated Pericytes Exhibit Increased Size and Morphological Complexity

**Fig. 1:**
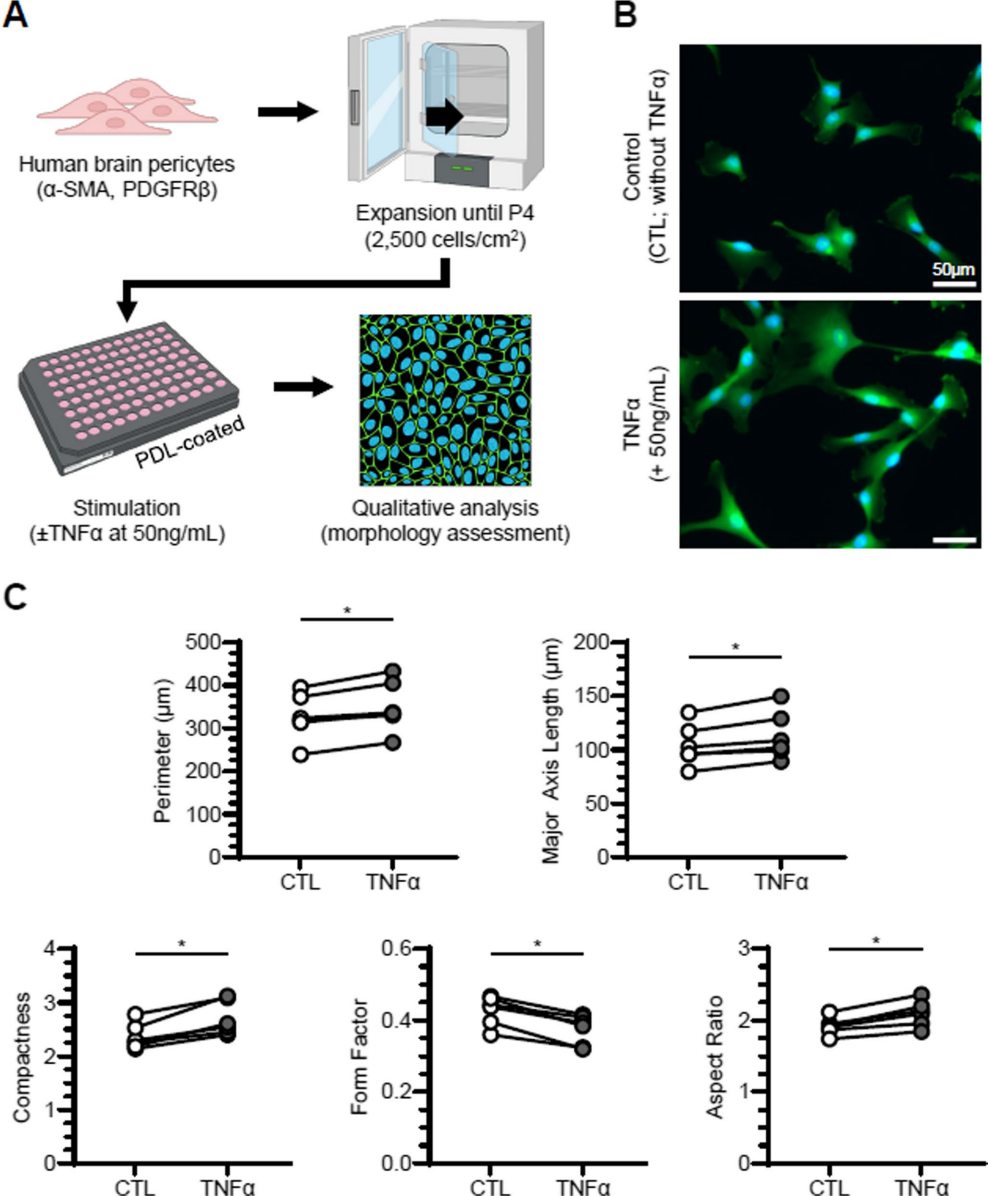
Pericytes become more complex and elongated with TNF-α stimulation. **A**. Schematic illustration of pericyte culture and their morphological assessment before and after TNF-α stimulation. **B** Representative images of unstimulated pericytes (CTL; top) and pericytes stimulated with 50ng/mL of TNF-α (TNF-α; bottom). Pericytes were stained with Hoechst (blue) and fluorescein-maleimide (green) to indicate nuclei and cytoplasm, respectively. **C** Quantification of morphological features between unstimulated (open circles) and stimulated pericytes (filled circles; *n* = 6 independent experiments). For each morphological feature, each data point represents an average of six replicate wells from a single experiment. *p < 0.05, Wilcoxon matched-pairs signed rank test

Human brain pericytes were expanded until passage 4, seeded into a 96-well plate coated with Poly-D-lysine (PDL) and treated with or without [50 ng/mL] TNF-α (Fig. 1A). Qualitatively, we observed that TNF-α-stimulated pericyte (TNF-α) morphology was different from that of unstimulated pericytes (CTL: Fig. 1B) as reflected in the significant and quantifiable change of multiple morphological features including perimeter, major axis length, compactness, form factor and aspect ratio (Fig. 1C). The stimulated pericytes increased significantly in perimeter and major axis length, illustrating an overall increase in size. A notable increase in aspect ratio also indicated a more elongated morphology upon stimulation, which coincided with the observed increase in major axis length. In addition, the stimulated pericytes decreased in form factor while increasing in compactness, which indicated an overall increase in complexity of morphology. Significant quantitative and qualitative changes in pericyte shape were consistently observed across six independent experiments, suggesting a robust morphological response to TNF-α stimulation.

### Immunological Stimulus Promotes Pro-inflammatory Secretory Phenotype in Pericytes

**Fig. 2:**
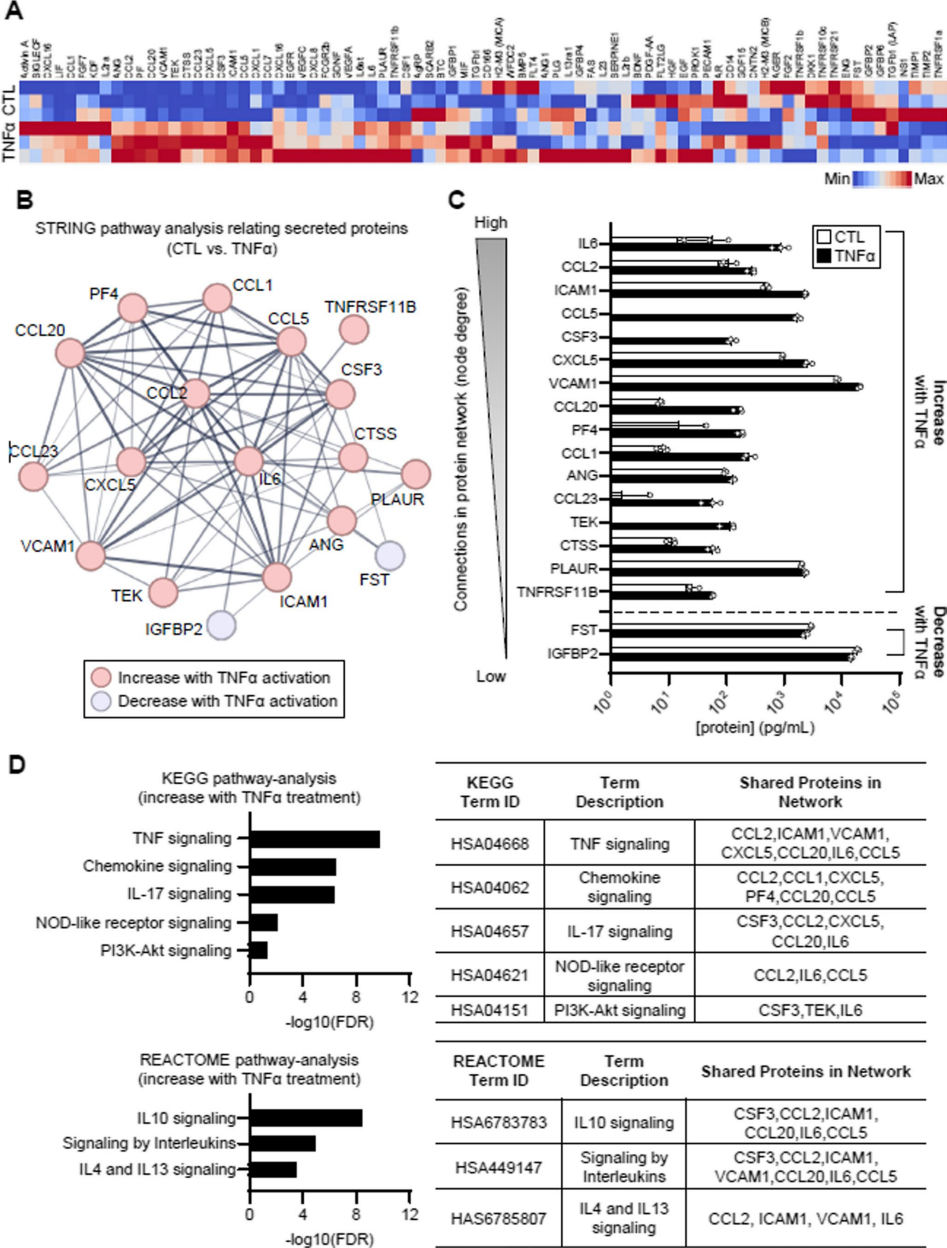
TNF-α stimulation promotes pro-inflammatory secretory phenotype in pericytes. **A** Heat map representing all the proteins secreted by unstimulated (CTL) and stimulated (TNF-α) pericytes. Two-way clustering performed using Ward method. Red = high secretion, Blue = Low secretion. *N* = 3 replicated conditioned medium samples per condition. **B** STRING pathway analysis relating secreted proteins. **C** Quantification of the secreted proteins after stimulation. Proteins are ordered from those with the most connections (node degrees) to the least number of connections within the network, and only the significantly changed proteins (*p* < 0.05) are shown. **D** Pathway enrichment analysis, KEGG and REACTOME, of the differentially secreted proteins. False discovery rate (log 10) of significant pathways is shown

To evaluate changes in protein secretory phenotype in addition to the morphological changes after immunological stimulation, we further performed quantitative secretome profiling on unstimulated and stimulated pericytes (Fig. 2). Pericytes were cultured on a 96-well plate with or without TNF-α stimulation, after which the supernatant was collected and analyzed using the Human Cytokine Array Q4000 (RayBiotech). 81 total proteins were detected as secreted by pericytes at levels above the pericyte medium-only control and were used for further analysis (Supplementary Table 2). Hierarchical clustering, which compared unstimulated to TNF-α-stimulated pericytes with three replicates in each group, illustrated a distinct secretion profile from pericytes after stimulation (Fig. 2A). Among 81 proteins, 18 proteins exhibited significant changes upon TNF-α stimulation, and further evaluation of these proteins using STRING analysis highlighted several proteins that were highly connected (based on node degree), such as IL-6, ICAM1, and CXCL10 (Fig. 2B). Proteins ordered by node degree, which indicates the number of connections in Fig. 2B, is shown in Supplementary Table 3. Among the 18 differentially expressed proteins, 16 were increased and 2 were decreased after TNF-α stimulation (Fig. 2C). Notably, the increased proteins included ICAM1 and VCAM1, which are relevant to pericyte physiology and morphology through mechanisms of adhesion, signaling, and response to inflammatory stimuli—indicating that immunological stimulation may alter cellular morphology and their physical function. Other increased proteins included pro-inflammatory chemokines CCL5, CSF3, CCL23, and TEK, which were below the threshold of detection in unstimulated groups but dramatically increased upon stimulation.

To better elucidate the signaling pathways associated with immunological stimulation in pericytes, we performed KEGG and REACTOME pathway analysis on the proteins that significantly increased upon TNF-α treatment (Fig. 2D). Our analysis identified multiple pathways related to chemokines and interleukins that were upregulated after treatment. IL-17 signaling significantly alters the inflammatory gene expression of pericytes [36] and has been shown to be a key driver in neuroinflammation [37, 38]. NOD-like pattern recognition receptors are expressed in pericytes, and activation of these receptors can lead to the secretion of inflammatory cytokines involved in neuroinflammation [39]. Similarly, PI3K-Akt signaling pathway in pericyte is also associated with extracellular matrix (ECM) degradation and BBB impairment by mediating VEGF expression [40]. These signaling pathways suggested how pericytes could play a mediator role in neuroinflammation. Pathways observed in stimulated pericytes included IL-4/IL-13 signaling, which involved both ICAM1 and VCAM1 in its protein network. Both neuroprotective and neurotoxic effects have been reported regarding IL-4/IL-13 signaling - whether these interleukins reduce inflammation or potentiate oxidative stress during neuroinflammation [41]. Indeed, enhanced expression of IL-4/IL-13 in activated microglia has been shown to play a critical role on neuronal death during Alzheimer’s disease [42]. Additionally, IL-4/IL-13 signaling is characteristic of the T helper type 2 (Th2) immune response, suggesting that an increase in this pathway is linked to eosinophil and other immune cell trafficking [43]. Overall, these data suggest that immunological stimulus of pericytes impacts the expression levels of proteins related to cellular physiology and morphology - such as ICAM1 and VCAM1 - which in turn may initiate downstream immune cascade involved in the regulation of neuroinflammation.

### MSC-EV Treatment Modulates Pericyte Morphology

**Fig. 3:**
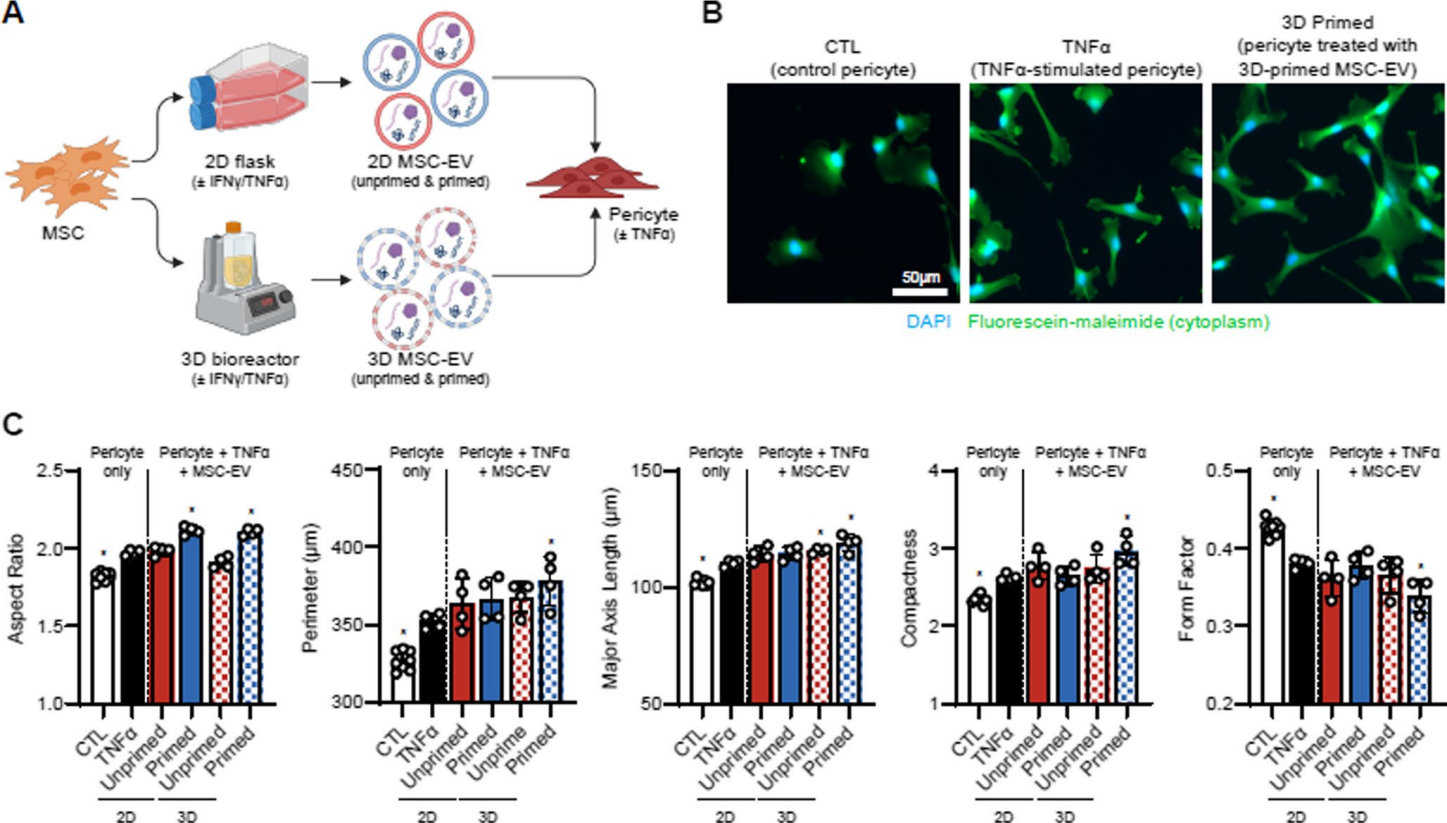
Cytokine priming of MSC-EVs enhances their impact on pericyte morphology. **A**. Schematic illustration of various MSC-EV manufacturing conditions. **B** Representative images of pericytes treated with different MSC-EVs from various conditions. Pericytes were stained with Hoechst (blue) and fluorescein-maleimide (green) to show nuclei and cytoplasm, respectively. **C** Quantification of morphological features of pericytes in response to various MSC-EV treatment. For each morphological feature, *n* = 6 wells per condition. *p < 0.05 relative to the stimulated pericyte group (TNFα). Data plotted as mean ± s.d

After establishing pericyte morphological and secretory control responses to TNF-α stimulation, we used this morphological assay to assess the effects of MSC-EVs produced under varying conditions. We treated pericytes with 4 different batches of MSC-EVs, generated from two different manufacturing platforms (2D-flasks vs. 500 mL vertical-wheel 3D-bioreactors), with or without 50 ng/mL IFN-γ/TNF-α priming (Fig. 3A). Cytokine priming is a well-established approach to mitigate functional heterogeneity of MSCs and enhance their immunomodulatory function [44]. These manufacturing conditions for MSC-EVs were adopted from a previous study by our group, which demonstrated significant modulation of microglia morphology and their secretome profile [2]. These MSC-EVs were characterized using the following assays for EV quality: protein (microBCA), size/count (Spectradyne), lipid bilayer and CD81 content (bead-based flow cytometry), morphology (TEM).

Interestingly, all MSC-EV groups demonstrated a general trend of increased size (perimeter, major axis length) and complexity (compactness, aspect ratio) in the stimulated pericytes—a trend also observed with immunological stimulation alone. Among the MSC-EV groups, those manufactured using IFN-γ/TNF-α priming in a bioreactor exhibited a consistent and significant changes in all morphological features, suggesting a robust effect of both the 3D culture platform and priming conditions (Fig. 3B, C). This highlighted that while TNF-α stimulation alone increased pericyte size, the addition of immunomodulatory MSC-EVs further enhanced this effect, indicating a complex relationship. In fact, this greater morphological change induced by MSC-EVs (i.e., ‘enhancement’ of morphological response to cytokines) in pericytes is contrary to our previous observation in microglia, where MSC-EV treatment resulted in a less pronounced morphological response (i.e. ‘suppression’ of morphological response), suggesting that MSC-EVs promoted an alternate morphological response for pericytes.

### Priming Condition and Microcarrier Concentration Impact MSC-EV Modulation of Pericyte Morphology

**Fig. 4:**
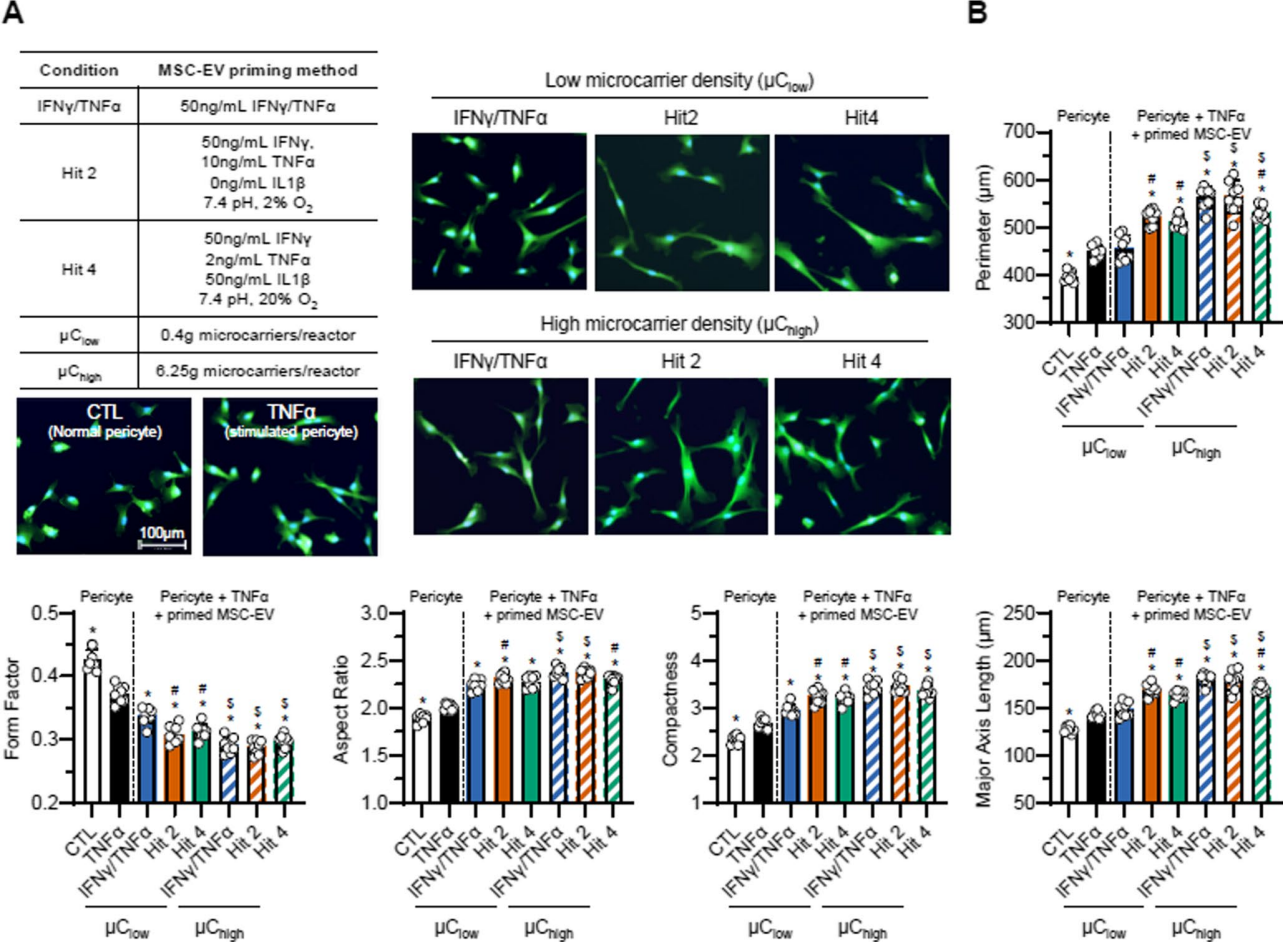
MSC-EV priming conditions and microcarrier density affect pericyte morphology. **A** Overview of MSC-EV manufacturing conditions (left) and representative images of pericytes cultured under these conditions (right). Pericytes were stained with Hoechst (blue) and fluorescein-maleimide (green) to show nuclei and cytoplasm, respectively.*** B,*** Quantification of morphological features of pericytes treated with MSC-EVs generated from various manufacturing conditions. For each morphological feature, *n* = 6 wells per condition. *p < 0.05 relative to the stimulated pericyte group (TNFα). ^#^*p* < 0.05 relative to the IFNγ/TNFα condition within µC density. ^$^*p* < 0.05 relative to µC_low_ within priming conditions

**Fig. 5:**
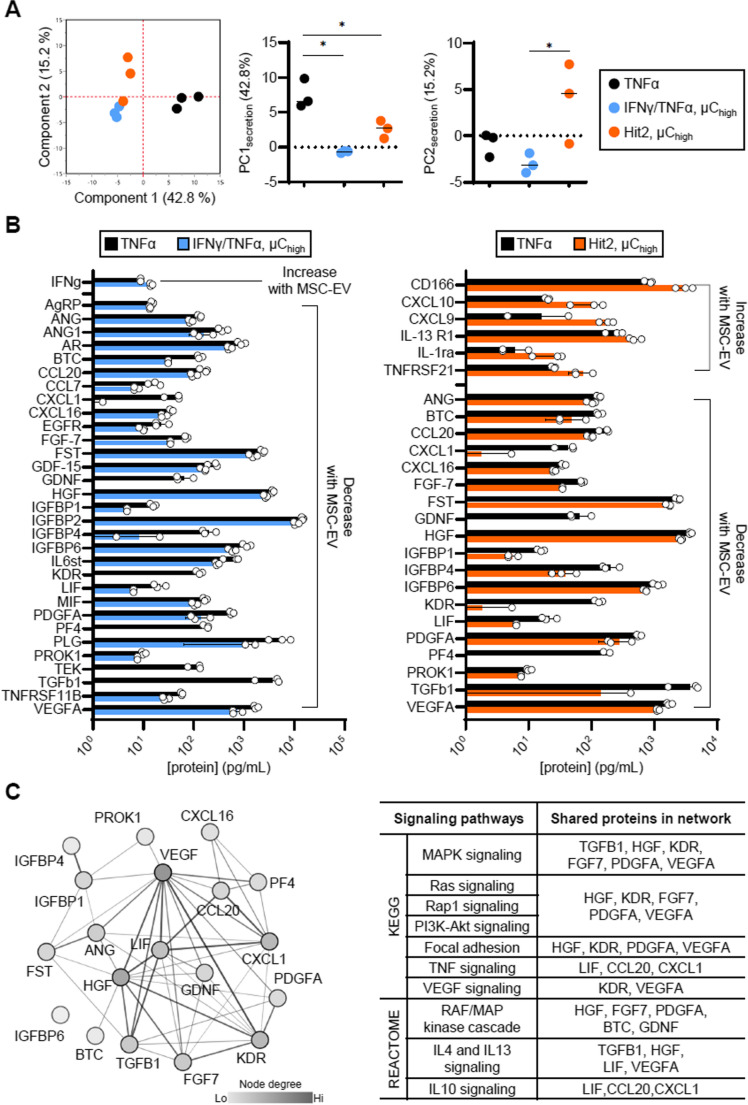
Primed MSC-EVs modulate pericyte secretome. **A** Individual clusters of TNFα (red), IFNγ/TNFα, µC_high_ (blue), and Hit2, µC_high_ (orange) observed with PCA conducted using all the proteins analyzed. *p < 0.05 using one-way ANOVA with Tukey’s multiple comparisons test across all groups. **B** Quantification of the pericyte proteins affected by MSC-EVs generated from IFNγ/TNFα, µC_high_ priming condition (left) or Hit 2, µC_high_ priming condition (right). **C** STRING network analysis (left) and KEGG/REACTOME pathway enrichment analysis (right) of downregulated proteins following MSC-EV treatment. The analysis focuses on proteins that were consistently decreased in response to IFNγ/TNFα and µC_high_ MSC-EV, as well as Hit 2, µC_high_ MSC-EV. For all bar graphs, data are mean ± s.d (*n* = 3 replicate conditioned medium sample per group). Only proteins significantly different between EV treatment groups and TNF-α control included as input to PCA in **A**, **B**

Changes in pericyte morphology may serve as indicators of underlying functional alterations, and morphology-associated changes can influence critical cell functions, impacting the regulation of inflammation and modulation of blood vessel formation. Therefore, we further explored broader manufacturing and priming conditions for MSC-EVs (Fig. 4), and profiled secretory phenotypes associated with these morphological changes (Fig. 5). For future studies, we chose to move forward with the bioreactor manufacturing platform based on several considerations: (1) The bioreactor primed group was the only MSC-EV group that consistently modulated pericyte morphology, and (2) bioreactors are amenable to scaling necessary for clinical production.

We explored additional manufacturing conditions that have previously shown more effective cell morphological modulation [2]. MSC-EVs were manufactured in the same 500 mL vertical wheel bioreactor using three different priming conditions and two different microcarrier concentrations with detailed descriptions of each condition tabulated in Fig. 4A. The IFN-γ/TNF-α priming condition is the same condition shown in Fig. 3A and the other 2 priming conditions (Hit 2 and Hit 4) were identified as ‘Morphological Hits’ from a high throughput morphological screen from our previous work and shown to have enhanced microglia modulatory activity versus unprimed MSCs [2].

We assessed the pericyte morphological response to these 6 different MSC-EV preparations using our established analysis pipeline. Similar to our previous observation (Fig. 3), we observed a notable difference between the TNF-α stimulated pericytes and all the MSC-EV groups, where MSC-EV treatments exhibited much bigger and elongated pericyte morphology (Fig. 4A). All MSC-EV groups significantly increased complexity (increase in aspect ratio and compactness, decrease in form factor) and size (increase in perimeter and major axis length) compared to the stimulated pericytes except for IFN-γ/TNF-α priming at low microcarrier concentration (µC_low_).

Furthermore, we were able to use pericyte morphology to assess the impact of cytokine priming and microcarrier conditions on MSC-EVs. Analysis of MSC-EV groups manufactured using µC_low_ showed Hit 2 and Hit 4 priming conditions had a significant effect on pericyte morphological response compared to IFN-γ/TNF-α priming (#*p* < 0.05, Fig. 4B). For MSC-EVs manufactured using high microcarrier (µC_high_) conditions, IFN-γ/TNF-α and Hit 2 priming groups were similar while Hit 4 group had a less significant enhancement effect on pericyte morphology, though still greater than the TNF-α-stimulated control. When comparing the role of microcarrier concentration, we found MSC-EV priming with IFN-γ/TNF-α or Hit 2 condition using µC_high_ enhanced pericyte morphological response more so than those using µC_low_, indicated by all morphological features ($*p* < 0.05, Fig. 4B). For Hit 4 priming conditions, this greater morphological response was only observed in perimeter, major axis length, compactness, and form factor, but not in aspect ratio.

### Primed MSC-EVs Modulate Secretory Phenotype From the Stimulated Pericytes

We then analyzed how two specific MSC-EV groups, from MSCs primed using IFN-γ/TNF-α or Hit 2 at µC_high_, affected pericyte secretome profile using a panel of 200 chemokines, cytokines and growth factors (Fig. 5). These 2 groups were chosen as they had the most significant impact on pericyte morphological response to TNF-α (Fig. 4). All proteins shown in Fig. 5 were detected at levels significantly above pericyte media (and pericyte media + MSC-EV only) controls indicating changes in secretion were from pericytes and not proteins from the MSC-EV prep.

Both IFN-γ/TNF-α and Hit 2 MSC-EV groups largely decreased the secretion of inflammatory cytokines, chemokines and growth factors– such as CXCL1, LIF, TGF β1, and VEGFA. A recent study demonstrated that immunological activation of pericytes significantly upregulated the production of these inflammatory secretome [45], and MSC-EVs generated from our primed batches were able to significantly regulate their secretion. This is particularly important because many of these secretome can induce pro-inflammatory states not just in pericytes, but also in astrocytes, microglia, and endothelial cells, and further recruit leukocytes to exacerbate neuroinflammation [46, 47]. Additionally, the expression of angiogenic factors, such as ANG, BTC, FGF-7, FST, GDNF, HGF, and VEGFA, have all decreased upon MSC-EV treatment in the stimulated pericytes. Neuroinflammation and angiogenesis are closely related process in the progression of CNS disorders, and while angiogenesis may help repair damaged tissue after inflammation, it also is a prominent feature of several CNS diseases [48, 49] as it can actively promote inflammation [50]. Indeed, injection of the well-recognized angiogenesis promoting factor VEGF into rat CNS was shown to induce both angiogenesis and inflammation [51]. Reduction of multiple angiogenic factors by MSC-EVs suggested a potential mechanism of action of our primed MSC-EV therapies in regulating neuroinflammation. Overall, enhanced secretion of immunological secretome after TNF- α stimulation in the pericytes were mitigated by both MSC-EV groups from different conditions.

Furthermore, there were several chemokines (CXCL9, CXCL10) and receptors (IL-1ra, IL-13R1, TNFRSF21, and CD166) that notably increased upon Hit 2 MSC-EV treatment. Both CXCL9 and CXCL10 are chemokines that are known to recruit activated T cells [52] and regulate vessel stability *via* CXCR3 receptor signaling pathways [53–55]. These chemokines can limit the function of endothelial cells during the resolving phase of wound healing [54, 56, 57], highlighting their importance in vascular remodeling during inflammation. Additionally, MSC-EV treatment greatly increased the levels of immunosuppressive membrane molecules IL-1ra [58, 59], IL-13R1 [60], and CD166 [61, 62] in pericytes, which all have immunomodulatory roles in T cell suppression, microglia activation, migration of pericytes, and tight junction permeability. Overall, these data underscore the regulatory role of MSC-EVs in modulating immune responses, while highlighting key secretome pathways that may contribute to neuroprotection through their beneficial effects on immune modulation and neural repair.

## Discussion

To fully realize the therapeutic potential of MSC-EVs, and facilitate their translation for neurodegenerative diseases, it is essential to develop robust assays that effectively demonstrate their immunomodulatory activity aligned with their proposed mechanisms of action. Here, we demonstrated how a high-throughput morphological screening bioassay can be applied to assess MSC-EV bioactivity on the target cell phenotype, pericytes. Our findings indicate that pericyte morphological responses are closely linked to the immunomodulatory activity of MSC-EVs across different production batches, particularly in the context of neurodegenerative disease treatment. Using this approach, we screened the effects of different manufacturing conditions on MSC-EV, and demonstrated that MSC-EVs enhanced the morphological response of pericytes, dramatically downregulating their neuroinflammatory secretome (e.g., VEGFA, TGFb1, CXCL1, CXCL16) and upregulating vascular remodeling secretory profile (e.g., CXCL9, CXCL10, IL-1ra, CD166).

Pericyte morphology is closely linked to their physiological roles in the context of neuroinflammation, with different morphologies associated with distinct functionalities [63]. For instance, one study suggested that elongated form of pericytes may increase the gap size between venules to promote neutrophil transmigration [64]. Another study demonstrated that bigger, and more elongated form of pericytes have reduced cellular motility and αSMA expression [65]. In our study, we observed consistent differences in both morphological (Fig. 1) and secretory phenotype (Fig. 2) between unstimulated pericytes and those stimulated with TNF-α. The morphological changes following stimulation included increases in cell size and complexity, as reflected by measures in perimeter, major axis length, aspect ratio, compactness, and a reduction in form factor. These results align with previous findings that TNF-α stimulation at 10ng/mL elongated the pericytes phenotype and enhanced their migratory behavior [66]. We believe this morphological change could be reflective of their function in vivo where they are known to migrate from sites of injury and hypoxia [67, 68]. In addition to morphological changes, TNF-α also influenced pericyte secretion profiles (Fig. 2). We observed a significant increase in IL-6, ICAM-1 and CCL5 expression in response to TNF-α, mirroring the findings from previous studies [69–71]. IL-6 has been shown to induce BBB dysregulation through the activation of microglia and astrocytes, leading to neuronal damage [69]. ICAM-1 and CCL5 both have roles in recruiting immune cells (i.e., monocytes, macrophages, NK cells, T cells) to the site of inflammation [72, 73]. Overall, our findings demonstrate TNF-α, a key mediator of neuroinflammation and several neurodegenerative diseases [31, 33], promotes a pro-inflammatory phenotype in pericytes in vitro. This suggests the pericytes may play an active role in recruiting both innate and adaptive immune cells to the site of inflammation in vivo, further supporting their involvement in neuroinflammatory processes.

In terms of the effect of EVs on pericyte morphological features, we initially hypothesized that EV treatment would suppress the pericyte morphological response to TNF-α (i.e. preventing their increase in size and complexity); however, MSC-EVs rather enhanced the effect in pericyte morphological response to TNF-α, suggesting larger size of pericytes does not necessarily equate to a more inflammatory form. This observation also contrasts with our previous study on microglia [74]. These pronounced morphological changes may reflect pericyte behaviors beyond immunomodulation, such as enhanced migration, cytoskeletal reorganization, differentiation, and vascular repair. Future studies investigating the relationship between these morphological changes and specific pericyte behaviors, particularly their effects on cells within the neurovascular unit, may reveal additional therapeutic mechanisms of MSC-EVs in neuroinflammatory conditions. MSC-EVs manufactured using 6 different conditions (Fig. 4A) increased the pericyte size (increase in perimeter, aspect ratio, and major axis length) and complexity (increase in compactness, and decrease in form factor), while significantly downregulating the secretion of pro-inflammatory secretome from the stimulated pericytes. Interestingly, these changes in cellular morphologies and secretory phenotypes have previously been seen in primed MSCs [27, 28]. Cytokine-primed MSCs, treated with either IFN-γ alone or a combination of IFN-γ and TNF-α, exhibited increase in size and complexity, while demonstrating an enhanced immunomodulatory function as seen in their T cell suppression. Although pericytes and MSCs possess different functional properties, they both share similar immunomodulatory activities in in vitro models [75, 76]. Considering the functional and phenotypic similarities observed between pericytes and MSCs both in vitro and in vivo, it is notable that they respond similarly to inflammatory signals (e.g. cytokines) in terms of their morphology. It is also important to note that the MSC-EVs can impact not only the pericytes themselves, but also their EV characteristics. A recent study has emphasized the emerging role of pericyte-derived EVs in vascular and neurological health [77]. Therefore, future studies elucidating how pericyte EVs are altered after immunological stimulation or therapeutic treatment are required.

Next, we hypothesized this alternate morphological response is indicative of pericytes having increased immunomodulatory function. We analyzed pericyte secretome in response to MSC-EVs as an indicator of MSC-EV bioactivity and found that MSC-EVs modulated pericyte secretion of proteins related to inflammatory response (Fig. 5). Among many proteins that decreased with MSC-EV treatment in pericytes, a significant reduction in angiogenic proteins, including ANG, BTC, TGF-β1, and VEGFA, was notable. The role of angiogenesis is context-dependent, and many studies have shown that neoangiogenesis can promote inflammation of the CNS [49, 78]. Among these proteins, BTC and VEGFA have shown direct correlation with neuroinflammation. Blocking Epidermal Growth Factor Receptor (EGFR), a receptor for a ligand BTC, can reduce microglial inflammatory response [79] and improve behavior changes in Alzheimer’s disease [80]. Similarly, a soluble receptor for VEGFA can reduce neuropathic pain development in nerve injury model [81]. Furthermore, our pathway analysis (Fig. 5C) demonstrated that MSC-EV treatments, regardless of their batch conditions from IFN-γ/TNF-α or Hit 2 priming, downregulated pathways associated with microglial immune response, such as MAPK and PI3K-AKT signaling [82, 83]. Collectively, these findings suggest that the primed MSC-EV therapies may indirectly modulate multiple cell-types in the BBB and brain parenchyma with pericytes serving as the key mediator.

MSC-EV manufacturing processes have been shown to change the functional outcomes of the EVs [2, 84, 85]. In this work, we observed that the magnitude of the enhanced pericyte morphological response was dependent on the manufacturing conditions. Compared to MSC-EVs produced by MSCs cultured in 2D flasks, MSCs cultured in 3D bioreactors produced EVs that consistently enhanced the pericyte morphological response to TNF-α i.e. increased size, elongation, and complexity (Fig. 3). Bioreactors are better suited for large scale manufacturing of MSC-EVs by enabling production of large, consistent batches of MSC-EVs and potentially mitigating the variability of multiple smaller MSC-EV batches produced using MSCs cultured in 2D flasks (or multilayered cellstacks). In addition, bioreactors allow for a dynamic environment that induces shear stress through stirring of the cell suspension that alters the topographical and surface environment the cells are exposed to, which can influence their behavior such as migration, adhesion, and morphology [86]. In our case, we found that the MSC-EVs produced with higher microcarrier densities had a greater effect on pericyte morphology, with a greater increase in pericyte size and complexity when compared to pericytes treated with MSC-EVs produced by MSCs cultured at lower microcarrier density. While the exact mechanism by which microcarrier density and priming impact MSC-EV bioactivity is unknown, it has been shown that the microcarrier concentration of 1.25 g per 90mL medium batch, which is similar to the high microcarrier density we have used in the experiment with 6.25 g per 500mL, yielded the highest MSC proliferation than that of the lower (0.75 g/90mL) or higher (2.5 g/90mL) microcarrier densities [87]. We observed that regardless of priming conditions, MSC-EVs in general exhibited a significant change in pericyte morphology, however, certain conditions (i.e., IFN-γ/TNF-α or Hit 2 priming) induced a greater change than the other (Hit 4 priming). Pericytes treated with IFN-γ/TNF-α MSC-EVs or Hit 2 MSC-EVs significantly secreted proteins that are associated with lipid response, such as CCL2, CXCL6, CXCL8, IL6, TNFRSF1B [2], and we have previously reported on the differences in the lipidomic profile of these various MSC-EV batches. Hence, further studies looking into batch-specific lipidomic signatures may enlighten the differential mechanisms of batch conditions on the pericyte morphologies.

## Conclusion

This is the first study demonstrating MSC-EV bioactivity in the context of modulating pericyte morphology and secretome profile. This novel approach provides a foundation for exploring MSC-EV potential in treating neurodegenerative diseases by using pericytes as an in vitro model of MSC-EV bioactivity. In addition, we believe that this assay will aid in standardization efforts that aim to assess MSC-EV bioactivity. Morphology is a rapid, single-cell, high throughout analysis that provides comprehensive information on cellular responses to disease-relevant signals such as inflammatory cytokines. The capabilities afforded by morphological profiling will facilitate standardization of MSC-EV characterization, evaluation of the effects of manufacturing changes on MSC-EV bioactivity, and further contribute to eventual clinical translation. While this study demonstrates the potential of using pericytes as a model target cell for assessing MSC-EV bioactivity relevant to treatment of neuroinflammation, it is important to note that the studies were conducted using pericytes derived from one donor and MSC-EVs produced from a single MSC line. This morphological platform can therefore be applied in the future to evaluate whether pericytes from different donors respond differently to MSC-EVs and whether MSC-EVs from different cell-lines (akin to different manufacturing conditions evaluated in this study) possess different bioactivity on the pericyte morphological response. Additionally, this work provides a foundation for exploring MSC-EV modulation of pericytes in more complex in vivo disease models or 3D in vitro systems [88, 89] (i.e. microphysiological ‘on-chip’ systems) that could improve our understanding of not only MSC-EV modulation of pericytes, but indirect modulation of other cell-types mediated by pericytes. Finally, this pericyte morphological assay could be used to better understand MSC-EV (or other cell-based therapy) mechanisms of action, the knowledge of which could help further refine MSC-EV manufacturing and facilitate clinical translation.

## Electronic Supplementary Material

Below is the link to the electronic supplementary material.

Supplementary Material 1

Supplementary Material 2

## Data Availability

No datasets were generated or analysed during the current study.

## References

[1] Avan, A., & Hachinski, V. (2021). Stroke and dementia, leading causes of neurological disability and death, potential for prevention. *Alzheimers Dement*, *17*(6), 1072–1076. 34057294 10.1002/alz.12340

[2] Larey, A. M., et al. (2024). High throughput screening of mesenchymal stromal cell morphological response to inflammatory signals for bioreactor-based manufacturing of extracellular vesicles that modulate microglia. *Bioact Mater*, *37*, 153–171. 38549769 10.1016/j.bioactmat.2024.03.009 PMC10972802

[3] Lyman, M., et al. (2014). Neuroinflammation: The role and consequences. *Neuroscience Research*, *79*, 1–12. 24144733 10.1016/j.neures.2013.10.004

[4] Alexander, J. J., et al. (2008). The complement cascade: Yin-Yang in neuroinflammation–neuro-protection and -degeneration. *Journal of Neurochemistry*, *107*(5), 1169–1187. 18786171 10.1111/j.1471-4159.2008.05668.x PMC4038542

[5] Ballabh, P., Braun, A., & Nedergaard, M. (2004). The blood-brain barrier: An overview: Structure, regulation, And clinical implications. *Neurobiology of Diseases*, *16*(1), 1–13. 10.1016/j.nbd.2003.12.016 15207256

[6] Nakano, M., et al. (2020). Bone marrow-derived mesenchymal stem cells improve cognitive impairment in an alzheimer’s disease model by increasing the expression of microRNA-146a in hippocampus. *Scientific Reports*, *10*(1), 10772. 32612165 10.1038/s41598-020-67460-1 PMC7330036

[7] Li, Y., et al. (2021). Three-dimensional cultured mesenchymal stem cells enhance repair of ischemic stroke through Inhibition of microglia. *Stem Cell Research & Therapy*, *12*(1), 358. 34154653 10.1186/s13287-021-02416-4 PMC8218508

[8] Achon Buil, B., Tackenberg, C., & Rust, R. (2023). Editing a gateway for cell therapy across the blood-brain barrier. *Brain*, *146*(3), 823–841. 36397727 10.1093/brain/awac393 PMC9976985

[9] Kallmeyer, K., et al. (2020). Fate of systemically and locally administered adipose-derived mesenchymal stromal cells and their effect on wound healing. *Stem Cells Transl Med*, *9*(1), 131–144. 31613054 10.1002/sctm.19-0091 PMC6954716

[10] Barkholt, L., et al. (2013). Risk of tumorigenicity in mesenchymal stromal cell-based therapies–bridging scientific observations and regulatory viewpoints. *Cytotherapy*, *15*(7), 753–759. 23602595 10.1016/j.jcyt.2013.03.005

[11] Tatsumi, K., et al. (2013). Tissue factor triggers procoagulation in transplanted mesenchymal stem cells leading to thromboembolism. *Biochemical and Biophysical Research Communications*, *431*(2), 203–209. 23313481 10.1016/j.bbrc.2012.12.134

[12] Ramos-Zaldivar, H. M., et al. (2022). Extracellular vesicles through the blood-brain barrier: A review. *Fluids Barriers CNS*, *19*(1), 60. 35879759 10.1186/s12987-022-00359-3 PMC9310691

[13] Brennan, M. A., Layrolle, P., & Mooney, D. J. (2020). Biomaterials functionalized with MSC secreted extracellular vesicles and soluble factors for tissue regeneration. *Advanced Functional Materials*, 30(37). 10.1002/adfm.201909125 PMC7494127 32952493

[14] Grapp, M., et al. (2013). Choroid plexus transcytosis and exosome shuttling deliver folate into brain parenchyma. *Nature Communications*, *4*(1), 2123. 23828504 10.1038/ncomms3123

[15] Yuan, O., et al. (2019). Exosomes derived from human primed mesenchymal stem cells induce mitosis and potentiate growth factor secretion. *Stem Cells and Development*, *28*(6), 398–409. 30638129 10.1089/scd.2018.0200 PMC6441283

[16] Lötvall, J. (2014). *Minimal experimental requirements for definition of extracellular vesicles and their functions: a position statement from the International Society for Extracellular Vesicles*. Wiley Online Library. p. 26913. 10.3402/jev.v3.26913 PMC4275645 25536934

[17] Kim, J. (2023). Clinical-Scale mesenchymal stem Cell-Derived extracellular vesicle therapy for wound healing. *International Journal of Molecular Sciences*, 24(5). 10.3390/ijms24054273 PMC10001880 36901703

[18] Neupane, Y. R., et al. (2023). Cell-derived nanovesicles from mesenchymal stem cells as extracellular vesicle-mimetics in wound healing. *Acta Pharm Sin B*, *13*(5), 1887–1902. 37250164 10.1016/j.apsb.2022.10.022 PMC10213815

[19] Ding, J. Y., et al. (2023). Mesenchymal stem cell-derived extracellular vesicles in skin wound healing: Roles, opportunities and challenges. *Mil Med Res*, *10*(1), 36. 37587531 10.1186/s40779-023-00472-w PMC10433599

[20] Fakouri, A., et al. (2024). Applications of mesenchymal stem cell-exosome components in wound infection healing: New insights. *Burns Trauma*, *12*, tkae021. 39139205 10.1093/burnst/tkae021 PMC11319788

[21] Harrell, C. R., et al. (2018). Molecular mechanisms underlying therapeutic potential of pericytes. *Journal of Biomedical Science*, *25*(1), 21. 29519245 10.1186/s12929-018-0423-7 PMC5844098

[22] Ahmed, T. A., & El-Badri, N. (2018). Pericytes: The role of multipotent stem cells in vascular maintenance and regenerative medicine. *Advances in Experimental Medicine and Biology*, *1079*, 69–86. 29282647 10.1007/5584_2017_138

[23] Cheng, J., et al. (2018). Targeting pericytes for therapeutic approaches to neurological disorders. *Acta Neuropathologica*, *136*(4), 507–523. 30097696 10.1007/s00401-018-1893-0 PMC6132947

[24] Rustenhoven, J., et al. (2017). Brain pericytes as mediators of neuroinflammation. *Trends in Pharmacological Sciences*, *38*(3), 291–304. 28017362 10.1016/j.tips.2016.12.001

[25] Yokota, K., et al. (2017). Periostin promotes Scar formation through the interaction between pericytes and infiltrating monocytes/macrophages after spinal cord injury. *American Journal of Pathology*, *187*(3), 639–653. 28082119 10.1016/j.ajpath.2016.11.010

[26] Reeves, C., et al. (2019). Spatiotemporal dynamics of PDGFRbeta expression in pericytes and glial Scar formation in penetrating brain injuries in adults. *Neuropathology and Applied Neurobiology*, *45*(6), 609–627. 30636077 10.1111/nan.12539 PMC6767497

[27] Klinker, M. W., et al. (2017). Morphological features of IFN-gamma-stimulated mesenchymal stromal cells predict overall immunosuppressive capacity. *Proc Natl Acad Sci U S A*, *114*(13), E2598–E2607. 28283659 10.1073/pnas.1617933114 PMC5380055

[28] Andrews, S. H., et al. (2022). Morphological landscapes from high content imaging reveal cytokine priming strategies that enhance mesenchymal stromal cell immunosuppression. *Biotechnology and Bioengineering*, *119*(2), 361–375. 34716713 10.1002/bit.27974

[29] Crisan, M., et al. (2008). A perivascular origin for mesenchymal stem cells in multiple human organs. *Cell Stem Cell*, *3*(3), 301–313. 18786417 10.1016/j.stem.2008.07.003

[30] Dore-Duffy, P., et al. (2006). CNS microvascular pericytes exhibit multipotential stem cell activity. *Journal of Cerebral Blood Flow and Metabolism*, *26*(5), 613–624. 16421511 10.1038/sj.jcbfm.9600272

[31] Xu, C., et al. (2021). TNF-alpha-dependent neuronal necroptosis regulated in alzheimer’s disease by coordination of RIPK1-p62 complex with autophagic UVRAG. *Theranostics*, *11*(19), 9452–9469. 34646380 10.7150/thno.62376 PMC8490500

[32] Vieira, M., et al. (2014). Ischemic insults induce necroptotic cell death in hippocampal neurons through the up-regulation of endogenous RIP3. *Neurobiology of Diseases*, *68*, 26–36. 10.1016/j.nbd.2014.04.002 24746856

[33] Shao, X., et al. (2020). TNF-alpha-induced p53 activation induces apoptosis in neurological injury. *Journal of Cellular and Molecular Medicine*, *24*(12), 6796–6803. 32344470 10.1111/jcmm.15333 PMC7299703

[34] Zhao, Z., et al. (2021). Isolation and analysis methods of extracellular vesicles (EVs). *Extracell Vesicles Circ Nucl Acids*, *2*(1), 80–103. 34414401 10.20517/evcna.2021.07 PMC8372011

[35] Carpenter, A. E., et al. (2006). CellProfiler: Image analysis software for identifying and quantifying cell phenotypes. *Genome Biology*, *7*(10), R100. 17076895 10.1186/gb-2006-7-10-r100 PMC1794559

[36] Liu, R., et al. (2016). IL-17 promotes Neutrophil-Mediated immunity by activating microvascular pericytes and not endothelium. *The Journal of Immunology*, *197*(6), 2400–2408. 27534549 10.4049/jimmunol.1600138 PMC5010945

[37] Chen, J., Liu, X., & Zhong, Y. (2020). Interleukin-17A: The key cytokine in neurodegenerative diseases. *Frontiers in Aging Neuroscience*, *12*, 566922. 33132897 10.3389/fnagi.2020.566922 PMC7550684

[38] Cao, Y., et al. (2021). IL (Interleukin)-17A acts in the brain to drive neuroinflammation, sympathetic activation, and hypertension. *Hypertension*, *78*(5), 1450–1462. 34628936 10.1161/HYPERTENSIONAHA.121.18219 PMC8516065

[39] Nyul-Toth, A., et al. (2017). Expression of pattern recognition receptors and activation of the non-canonical inflammasome pathway in brain pericytes. *Brain, Behavior, and Immunity*, *64*, 220–231. 28432035 10.1016/j.bbi.2017.04.010

[40] Geranmayeh, M. H., Rahbarghazi, R., & Farhoudi, M. (2019). Targeting pericytes for neurovascular regeneration. *Cell Commun Signal*, *17*(1), 26. 30894190 10.1186/s12964-019-0340-8 PMC6425710

[41] Mori, S., Maher, P., & Conti, B. (2016). Neuroimmunology of the Interleukins 13 and 4. *Brain Sciences* 6(2). 10.3390/brainsci6020018 PMC4931495 27304970

[42] Jeong, J. Y., Chung, Y. C., & Jin, B. K. (2019). Interleukin-4 and interleukin-13 exacerbate neurotoxicity of prothrombin Kringle-2 in cortex in vivo via oxidative stress. *International Journal of Molecular Sciences*, *20*(8). 10.3390/ijms20081927 10.3390/ijms20081927 PMC6515094 31010119

[43] Garrison, A. T., et al. (2023). Pericytes: The lung-forgotten cell type. *Frontiers in Physiology*, *14*, 1150028. 37035669 10.3389/fphys.2023.1150028 PMC10076600

[44] Kouroupis, D., et al. (2019). Mesenchymal stem cell functionalization for enhanced therapeutic applications. *Tissue Eng Part B Rev*, *25*(1), 55–77. 30165783 10.1089/ten.TEB.2018.0118

[45] Kaushik, D. K., et al. (2021). Pericytes as mediators of infiltration of macrophages in multiple sclerosis. *J Neuroinflammation*, *18*(1), 301. 34952601 10.1186/s12974-021-02358-x PMC8705458

[46] Takata, F., et al. (2011). Brain pericytes among cells constituting the blood-brain barrier are highly sensitive to tumor necrosis factor-alpha, releasing matrix metalloproteinase-9 and migrating in vitro. *J Neuroinflammation*, *8*, 106. 21867555 10.1186/1742-2094-8-106 PMC3182916

[47] Kovac, A., Erickson, M. A., & Banks, W. A. (2011). Brain microvascular pericytes are immunoactive in culture: Cytokine, chemokine, nitric oxide, and LRP-1 expression in response to lipopolysaccharide. *J Neuroinflammation*, *8*, 139. 21995440 10.1186/1742-2094-8-139 PMC3207972

[48] Seabrook, T. J., et al. (2010). Angiogenesis is present in experimental autoimmune encephalomyelitis and pro-angiogenic factors are increased in multiple sclerosis lesions. *J Neuroinflammation*, *7*, 95. 21176212 10.1186/1742-2094-7-95 PMC3022818

[49] Shahriar, S., et al. (2024). VEGF-A-mediated venous endothelial cell proliferation results in neoangiogenesis during neuroinflammation. *Nature Neuroscience*, *27*(10), 1904–1917. 39256571 10.1038/s41593-024-01746-9

[50] Muramatsu, R., et al. (2012). Angiogenesis induced by CNS inflammation promotes neuronal remodeling through vessel-derived Prostacyclin. *Nature Medicine*, *18*(11), 1658–1664. 23042236 10.1038/nm.2943

[51] Croll, S. D., et al. (2004). VEGF-mediated inflammation precedes angiogenesis in adult brain. *Experimental Neurology*, *187*(2), 388–402. 15144865 10.1016/j.expneurol.2004.02.010

[52] Michlmayr, D., & McKimmie, C. S. (2014). Role of CXCL10 in central nervous system inflammation. *International Journal of Interferon Cytokine and Mediator Research*, *6*, 1–18. 10.2147/IJICMR.S35953

[53] Bodnar, R. J., et al. (2013). Pericyte regulation of vascular remodeling through the CXC receptor 3. *Arteriosclerosis, Thrombosis, and Vascular Biology*, *33*(12), 2818–2829. 24135023 10.1161/ATVBAHA.113.302012 PMC4591000

[54] Zaja-Milatovic, S., & Richmond, A. (2008). CXC chemokines and their receptors: A case for A significant biological role in cutaneous wound healing. *Histology and Histopathology*, *23*(11), 1399–1407. 18785122 10.14670/hh-23.1399 PMC3140405

[55] Bodnar, R. J., Yates, C. C., & Wells, A. (2006). IP-10 blocks vascular endothelial growth factor-induced endothelial cell motility and tube formation via Inhibition of Calpain. *Circ Res*, *98*(5), 617–625. 16484616 10.1161/01.RES.0000209968.66606.10 PMC3826264

[56] Barrientos, S., et al. (2008). Growth factors and cytokines in wound healing. *Wound Repair and Regeneration: Official Publication of the Wound Healing Society [And] the European Tissue Repair Society*, *16*(5), 585–601. 10.1111/j.1524-475X.2008.00410.x 19128254

[57] Yates, C. C., et al. (2008). ELR-negative CXC chemokine CXCL11 (IP-9/I-TAC) facilitates dermal and epidermal maturation during wound repair. *American Journal of Pathology*, *173*(3), 643–652. 18669615 10.2353/ajpath.2008.070990 PMC2527079

[58] To, X. V., et al. (2023). Anti-inflammatory Interleukin 1 receptor antagonist concentration in plasma correlates with blood-brain barrier integrity in the primary lesion area in traumatic brain injury patients. *Brain Behav Immun Health*, *31*, 100653. 37415924 10.1016/j.bbih.2023.100653 PMC10320227

[59] Valdor, R., et al. (2017). Glioblastoma progression is assisted by induction of immunosuppressive function of pericytes through interaction with tumor cells. *Oncotarget*, *8*(40), 68614. 28978142 10.18632/oncotarget.19804 PMC5620282

[60] Li, S., et al. (2023). Interleukin-13 and its receptor are synaptic proteins involved in plasticity and neuroprotection. *Nature Communications*, *14*(1), 200. 36639371 10.1038/s41467-023-35806-8 PMC9839781

[61] Lecuyer, M. A., et al. (2017). Dual role of ALCAM in neuroinflammation and blood-brain barrier homeostasis. *Proc Natl Acad Sci U S A*, *114*(4), E524–E533. 28069965 10.1073/pnas.1614336114 PMC5278491

[62] Cho, W. J., Mittal, S. K., & Chauhan, S. K. (2023). Mesenchymal stromal cells suppress T-Cell-Mediated Delayed-Type hypersensitivity via ALCAM-CD6 interaction. *Stem Cells Transl Med*, *12*(4), 221–233. 36972356 10.1093/stcltm/szad012 PMC10108723

[63] Alarcon-Martinez, L., Yemisci, M., & Dalkara, T. (2021). Pericyte morphology and function. *Histology and Histopathology*, *36*(6), 633–643. 33595091 10.14670/HH-18-314

[64] Proebstl, D., et al. (2012). Pericytes support neutrophil subendothelial cell crawling and breaching of venular walls in vivo. *Journal of Experimental Medicine*, *209*(6), 1219–1234. 22615129 10.1084/jem.20111622 PMC3371725

[65] Brown, L. S., et al. (2023). Brain pericytes in culture display diverse morphological and functional phenotypes. *Cell Biology and Toxicology*, *39*(6), 2999–3014. 37322257 10.1007/s10565-023-09814-9 PMC10693527

[66] Tigges, U., et al. (2013). TNF-alpha promotes cerebral pericyte remodeling in vitro, via a switch from alpha1 to alpha2 integrins. *J Neuroinflammation*, *10*, 33. 23448258 10.1186/1742-2094-10-33 PMC3616978

[67] Dore-Duffy, P., et al. (2000). Pericyte migration from the vascular wall in response to traumatic brain injury. *Microvascular Research*, *60*(1), 55–69. 10873515 10.1006/mvre.2000.2244

[68] Gonul, E., et al. (2002). Early pericyte response to brain hypoxia in cats: An ultrastructural study. *Microvascular Research*, *64*(1), 116–119. 12074637 10.1006/mvre.2002.2413

[69] Matsumoto, J., et al. (2018). TNF-alpha-sensitive brain pericytes activate microglia by releasing IL-6 through Cooperation between IkappaB-NFkappaB and JAK-STAT3 pathways. *Brain Research*, *1692*, 34–44. 29702085 10.1016/j.brainres.2018.04.023

[70] Persidsky, Y., et al. (2016). Dysfunction of brain pericytes in chronic neuroinflammation. *Journal of Cerebral Blood Flow and Metabolism*, *36*(4), 794–807. 26661157 10.1177/0271678X15606149 PMC4821019

[71] Matsumoto, J., et al. (2014). Tumor necrosis factor-alpha-stimulated brain pericytes possess a unique cytokine and chemokine release profile and enhance microglial activation. *Neuroscience Letters*, *578*, 133–138. 24993300 10.1016/j.neulet.2014.06.052

[72] Smyth, L. C. D., et al. (2018). Unique and shared inflammatory profiles of human brain endothelia and pericytes. *J Neuroinflammation*, *15*(1), 138. 29751771 10.1186/s12974-018-1167-8 PMC5948925

[73] Griffith, J. W., Sokol, C. L., & Luster, A. D. (2014). Chemokines and chemokine receptors: Positioning cells for host defense and immunity. *Annual Review of Immunology*, *32*, 659–702. 24655300 10.1146/annurev-immunol-032713-120145

[74] Daga, K. R., et al. (2024). Microglia morphological response to mesenchymal stromal cell extracellular vesicles demonstrates EV therapeutic potential for modulating neuroinflammation. *J Biol Eng*, *18*(1), 58. 39420399 10.1186/s13036-024-00449-w PMC11488223

[75] Blocki, A., et al. (2013). Not all MSCs can act as pericytes: Functional in vitro assays to distinguish pericytes from other mesenchymal stem cells in angiogenesis. *Stem Cells and Development*, *22*(17), 2347–2355. 23600480 10.1089/scd.2012.0415 PMC3749721

[76] da Silva Meirelles, L., et al. (2016). Mesenchymal stem cells and their relationship to pericytes. *Front Biosci (Landmark Ed)*, *21*(1), 130–156. 26709765 10.2741/4380

[77] Sharma, K. (2022). The Emerging Role of Pericyte-Derived Extracellular Vesicles in Vascular and Neurological Health. *Cells* 11(19). 10.3390/cells11193108 PMC9563036 36231071

[78] Kirk, S. L., & Karlik, S. J. (2003). VEGF and vascular changes in chronic neuroinflammation. *Journal of Autoimmunity*, *21*(4), 353–363. 14624758 10.1016/s0896-8411(03)00139-2

[79] Qu, W. S., et al. (2012). Inhibition of EGFR/MAPK signaling reduces microglial inflammatory response and the associated secondary damage in rats after spinal cord injury. *J Neuroinflammation*, *9*, 178. 22824323 10.1186/1742-2094-9-178 PMC3418570

[80] Mansour, H. M., et al. (2022). Repurposed anti-cancer epidermal growth factor receptor inhibitors: Mechanisms of neuroprotective effects in alzheimer’s disease. *Neural Regen Res*, *17*(9), 1913–1918. 35142667 10.4103/1673-5374.332132 PMC8848623

[81] Peng, Z., et al. (2022). Targeting vascular endothelial growth factor A with soluble vascular endothelial growth factor receptor 1 ameliorates nerve injury-induced neuropathic pain. *Molecular Pain*, *18*, 17448069221094528. 35354377 10.1177/17448069221094528 PMC9706061

[82] Bachstetter, A. D., et al. (2011). Microglial p38alpha MAPK is a key regulator of Proinflammatory cytokine up-regulation induced by toll-like receptor (TLR) ligands or beta-amyloid (Abeta). *J Neuroinflammation*, *8*, 79. 21733175 10.1186/1742-2094-8-79 PMC3142505

[83] He, X., et al. (2022). The PI3K/AKT signalling pathway in inflammation, cell death and glial Scar formation after traumatic spinal cord injury: Mechanisms and therapeutic opportunities. *Cell Proliferation*, *55*(9), e13275. 35754255 10.1111/cpr.13275 PMC9436900

[84] Andrews, S., et al. (2021). Priming of MSCs with inflammation-relevant signals affects extracellular vesicle biogenesis, surface markers, and modulation of T cell subsets. *Journal of Immunology and Regenerative Medicine*, *13*, 100036.

[85] Hackel, A., et al. (2023). Immunological priming of mesenchymal stromal/stem cells and their extracellular vesicles augments their therapeutic benefits in experimental graft-versus-host disease via engagement of PD-1 ligands. *Frontiers in Immunology*, *14*, 1078551. 36875112 10.3389/fimmu.2023.1078551 PMC9978482

[86] Hupfeld, J., et al. (2014). Modulation of mesenchymal stromal cell characteristics by microcarrier culture in bioreactors. *Biotechnology and Bioengineering*, *111*(11), 2290–2302. 24890974 10.1002/bit.25281

[87] Lembong, J. (2020). Bioreactor parameters for Microcarrier-Based human MSC expansion under Xeno-Free conditions in a Vertical-Wheel system. *Bioengineering (Basel)*, *7*(3 ), 73. 10.3390/bioengineering7030073 10.3390/bioengineering7030073 PMC7552727 32650422

[88] Kwee, B. J., et al. (2023). Modeling immunity in microphysiological systems. *Exp Biol Med (Maywood)*, *248*(22), 2001–2019. 38166397 10.1177/15353702231215897 PMC10800123

[89] Lam, J., et al. (2024). Characterizing On-Chip angiogenesis induction in a microphysiological system as a functional measure of mesenchymal stromal cell bioactivity. *Adv Biol (Weinh)*, *8*(8), e2300094. 37409400 10.1002/adbi.202300094

