# Supplementary material for: Development of a High-throughput Morphological Assay for Evaluating Mesenchymal Stromal Cell-derived Extracellular Vesicle Modulation of Brain Pericyte Secretory Phenotype
Source: Stem Cell Rev Rep. 2025 Aug 12;21(8):2781–95. doi: 10.1007/s12015-025-10940-6 (PMC12504408; doi:10.1007/s12015-025-10940-6)
Supplement: Supplementary file 1 — Supplementary Material 1 [file 12015_2025_10940_MOESM1_ESM.pdf]

Measurements made by CellProfiler	Description
Perimeter	The total number of pixels around the boundary of each region in the image.
Major Axis Length	The length of the major axis of the ellipse that has the same normalized second central moments as the region.
Compactness	Calculated as $Perimeter^2/4*\pi*Area$, related to Form Factor. A filled circle will have a compactness of 1, with irregular objects or objects with holes having a value greater than 1.
Form Factor	Calculated as $4*\pi*Area/Perimeter^2$. Equals 1 for a perfectly circular object.
Aspect Ratio	Major axis length divided by minor axis length.

Supplementary Table 1. Table defining 5 morphological features quantified in the study using CellProfiler.

Proteins detected as secreted by pericytes at levels above the pericyte medium-only control								
Activin A	BMP-5	EGF	CSF3	IGFBP1	CXCL8	MICB	PF4	TNF RII
AgRP	BTC	EGF R	CXCL6	IGFBP2	INS	MIF	MOK	TRAIL R3
ALCAM	CTSS	CXCL5	GDF15	IGFBP4	LAP(TGFb1)	CCL20	CCL5	PI3
ANG1	CD14	ENG	GDNF	IGFBP6	LIF	CCL23	Siglec-5	PLAUR
ANG	CNTN2	FAS	LRPPRC	IL-13 R1	SCARB2	NTF4	TGFb1	VCAM-1
PLG	CXCL16	Fcg RIIBC	CXCL1	IL-2 Ra	CCL2	TNFRSF11B	TEK	VEGF
AR	DKK-1	FGF-7	HGF	IL-2 Rb	CCL7	SERPINE1	TIMP-1	VEGF R2
BDNF	TNFRSF21	Flt-3L	CCL1	IL-23	CSF1	PDGF-AA	TIMP-2	VEGF R3
bFGF	PROK1	FST	ICAM-1	IL-6	MICA	PECAM-1	TNF RI	VEGF-C

Supplementary Table 2. Comprehensive list of proteins secreted by pericytes detected above levels in pericyte medium-only control.

Protein	Identifier	Node degree	CTL_1	CTL_2	CTL_3	TNF-α_1	TNF-α_2	TNF-α_3
IL6	9606.ENSP00000385675	17	51.3	108.8	18.5	869.0	617.7	1,205.6
CCL2	9606.ENSP00000225831	14	83.5	151.8	88.9	227.4	300.5	304.7
ICAM1	9606.ENSP00000264832	12	501.8	427.8	553.1	2,438.2	2,303.9	2,340.6
CCL5	9606.ENSP00000474412	11	0.0	0.0	0.0	1,694.8	1,946.0	1,545.5
CSF3	9606.ENSP00000225474	11	0.0	0.0	0.0	107.3	148.6	118.5
CXCL5	9606.ENSP00000296027	11	983.0	917.5	934.5	2,323.7	3,099.7	2,419.8
VCAM1	9606.ENSP00000294728	11	7,895.7	8,900.0	8,055.6	18,642.6	20,989.2	21,301.6
CCL20	9606.ENSP00000351671	10	7.8	6.5	7.3	131.4	185.0	176.2
PF4	9606.ENSP00000296029	10	0.0	45.0	0.0	154.8	195.2	192.8
CCL1	9606.ENSP00000225842	8	8.2	9.5	6.1	314.0	203.2	218.2
ANG	9606.ENSP00000336762	7	93.3	100.8	90.5	121.1	135.2	139.6
CCL23	9606.ENSP00000481357	7	0.0	4.8	0.0	57.5	79.2	36.3
TEK	9606.ENSP00000369375	6	0.0	0.0	0.0	76.5	130.5	134.6
CTSS	9606.ENSP00000357981	5	12.5	12.9	9.1	58.9	70.7	47.4
PLAUR	9606.ENSP00000339328	4	2,066.5	1,924.2	2,043.6	2,276.6	2,286.7	2,457.2
TNFRSF11B	9606.ENSP00000297350	1	22.5	23.1	34.1	58.2	55.3	60.2
FST	9606.ENSP00000256759	3	2,840.2	3,045.0	2,925.6	2,426.1	2,141.8	2,542.8
IGFBP2	9606.ENSP00000233809	2	17,001.1	20,017.9	18,614.4	15,231.4	14,005.5	14,477.5

Supplementary Table 3. Comprehensive list of upregulated (orange) and downregulated (grey) proteins after TNF-α stimulation in pericytes. Proteins are ordered by the node degree.
